# Dreidimensionale Planung und Durchführung von Hüft- und Beckeneingriffen

**DOI:** 10.1007/s00132-026-04823-y

**Published:** 2026-04-24

**Authors:** Selina Streich, Domenic Grisch, Thomas Dreher

**Affiliations:** 1 Department Pediatric Orthopedics and Traumatology, University Children’s Hospital Zurich, Zürich, Schweiz; 2 Department of Orthopedic Surgery, University Hospital Balgrist, University of Zurich, Zürich, Schweiz

**Keywords:** 3‑D-Bildgebung, Kinder, Lebensqualität, Revisionschirurgie, Behandlungsergebnis, 3‑D imaging, Children, Quality of Life, Revision surgery, Treatment outcome

## Abstract

**Hintergrund:**

Eine Vielzahl unterschiedlicher Erkrankungen in allen Altersgruppen, von Kleinkindern bis zu Erwachsenen, erfordert operative Eingriffe an Hüfte und Becken. Gut etablierte Techniken sind in Planung und Durchführung komplex und basieren derzeit größtenteils auf zweidimensionaler prä- und intraoperativer Bildgebung. Dies kann zu potenzieller Ungenauigkeit und hoher Outcome-Variabilität führen. Unzureichende Korrekturen bergen ein erhebliches Risiko für Rezidivbeschwerden, welche möglicherweise eine Re-Operation erforderlich machen.

**Fallbeispiele:**

In dieser Fallserie demonstrieren wir anhand von drei eindrucksvollen Beispielen die Chancen des Einsatzes dreidimensionaler Bildgebung und derer Anwendungsmöglichkeiten in der Diagnostik, präoperativen Planung und zur intraoperativen Orientierung durch Navigation oder Guides bei Routineeingriffen sowie bei komplexen Fällen. Alle Fälle zeigten präzise Korrekturen und zufriedenstellende postoperative Verläufe.

**Schlussfolgerungen:**

Zur Minimierung von Ungenauigkeiten und zum breiteren Einsatz in der Klinik schlagen wir daher weitere Forschung vor, um standardisierte Protokolle für den Einsatz dieser unterstützenden Modalitäten zu entwickeln. Das geschieht mit der Intension, das Risiko für ein Rezidiv oder eine frühzeitige Koxarthrose zu mindern und damit die längerfristige gesundheitsbezogene Lebensqualität im Sinne einer patientenspezifischen Medizin zu verbessern. Aufgrund der bislang fehlenden jeweils spezifischen Datenlage bleibt für den breiten Einsatz in der klinischen Routine die Präzisionssteigerung und deren langfristiger Einfluss aufzuzeigen.

## Abstract (en)

**Background:**

Different conditions among all age groups, from toddlers to adults, require surgical procedures at the hip and pelvis. Well-established techniques are complex in planning and execution and currently rely mostly on two-dimensional pre- and intraoperative imaging, resulting in potential imprecision and outcome variability. Inadequate correction carries a considerable risk for recurring symptoms requiring revision surgery.

**Case examples:**

In this case series, we present three patient examples using three-dimensional imaging and their application to explore the chances these modalities offer for pre-operative planning and intraoperative referencing and navigation in routine and complex interventions. All three cases showed precise corrections and satisfactory postoperative follow-ups.

**Conclusions:**

To minimize inaccuracies and enable broader use in clinical practice, we therefore recommend further research to develop standardized protocols for the use of these supportive modalities with the aim of reducing the risk of recurrence or early coxarthrosis and thus improving long-term health-related quality of life in the spirit of patient-specific medicine. Due to the lack of specific data to date, the increase in precision and its long-term impact still need to be demonstrated for widespread use in routine clinical practice.

Die Planung operativer Eingriffe basiert auf etablierten, aber oftmals komplexen Techniken. Da diese größtenteils auf zweidimensionaler prä- und intraoperativer Bildgebung basieren, besteht die Gefahr von Ungenauigkeiten und einer hohen Variabilität im Outcome mit Rezidiven. Der Einsatz der dreidimensionalen Bildgebung kann helfen, diese Ungenauigkeiten deutlich zu reduzieren und das Outcome verbessern.

## Einleitung

Eine Vielzahl unterschiedlicher Erkrankungen in allen Altersgruppen erfordert rekonstruktive Eingriffe an Hüfte und Becken. Im Kindes- und Jugendalter sind dies unter anderem die kongenitale („developmental dysplasia of the hip“ [DDH]) oder sekundäre entwicklungsbedingte Hüftgelenksdysplasie und das femoroazetabuläre Impingement (FAI) [1]. Bei der DDH besteht eine angeborene oder, beispielsweise bei Kindern mit Zerebralparese (CP), sekundär durch muskuläre Imbalance und Torsionsdeformitäten bedingte unzureichende azetabuläre Überdachung mit Instabilität und (Sub‑)Luxation des Femurkopfs. Während die kongenitale Form bis zu 6 % aller Neugeborenen betrifft, ist es jedes dritte Kind mit CP, wobei die Prävalenz mit dem Grad der funktionellen motorischen Einschränkung (Gross Motor Functions Classification System [GMFCS]) korreliert und bei Kindern in den GMFCS-Stufen IV–V über 50 % beträgt [2, 3]. Beim FAI kommt es zu einem mechanischen Konflikt aufgrund von Fehlstellungen des Azetabulums, beispielsweise Retrotorsion, und Morphologieveränderungen am proximalen Femur, wie dies im Rahmen fehlverheilter Epiphyseolysis capitis femoris, Morbus Perthes, Trauma oder Überbelastung möglich ist [4]. Beides verursacht durch mechanische Fehlbelastung und Reizung Schmerzen, Funktionseinschränkungen und vorzeitige Hüftarthrose [5]. Zur Vermeidung dieser Folgeschäden ist das Wiedererlangen einer physiologischen Lastübertragung und regelrechten Gelenkskongruenz wegweisend.

Die Wahl der rekonstruktiven Beckenosteotomien variiert je nach Alter und Deformitätsmuster und unterscheidet Umformungs- von Umstellungsverfahren [6]. Die Azetabuloplastik nach Pemberton oder Dega korrigiert eine fehlende Kongruenz durch eine unvollständige Umformungsosteotomie des Iliums über der Y‑Fuge mit Einsetzen eines Knochenkeils [7]. Ist die Hüftgelenkskongruenz erhalten, sind Umstellungsosteotomien zu erwägen: Bei Kindern unter 8 Jahren kann ein zu steiles Azetabulum mittels vollständiger Ilium-Osteotomie nach Salter um die Symphyse rotiert und eine anterolaterale Mehrüberdachung erreicht werden. Die Triple-Osteotomie der Ossa ilium, pubis und ischii nach Tönnis erlaubt die multidirektionale Reorientierung der Pfanne im Adoleszentenalter (8–14 Jahre) bei nahezu geschlossener Y‑Fuge [8]. Mit dem Schluss der Y‑Fuge (ab 14 Jahren) ermöglicht die periazetabuläre Osteotomie (PAO) nach Ganz eine präzise multidirektionale Korrektur [9]. Häufig besteht zudem eine femorale Fehlstellung im Sinne einer Coxa valga et antetorta, weshalb die Eingriffe mit varisierenden, derotierenden proximalen Femurosteotomien (VDRO) kombiniert werden [10]. Bei allen Vorgehen werden die Osteotomieebenen und Einstellungskorrekturen freihändig unter intraoperativer Durchleuchtungskontrolle (Bildverstärker [BV]) durchgeführt.

Alle Verfahren sind technisch anspruchsvoll und erfordern eine präzise Planung

Alle Verfahren sind technisch anspruchsvoll und erfordern eine präzise Planung sowie hohe chirurgische Expertise, um langfristig zufriedenstellende Ergebnisse zu erzielen. Typische Komplikationen umfassen unmittelbare Risiken wie Blutverlust, lange Operationszeiten, iatrogene Knorpel- oder Labrumschädigungen sowie neurovaskuläre Verletzungen. Über- oder Unterkorrekturen sowie Fragmentfehlstellungen oder -rotationen bergen das Risiko von Reoperation aufgrund insuffizienter Beschwerdelinderung und zur langfristigen Prävention von Folgebeschwerden. Steppacher et al. (2008) konnte aufzeigen, dass eine Residualdysplasie nach PAO mit frühzeitiger Arthrose und erhöhtem Endoprothesenbedarf assoziiert ist [11]. In der Erwachsenenpopulation zeigte Novais et al. (2016), dass die radiologischen Zielparameter bei PAO-Korrektur auch bei erfahrenen Chirurgen nur zu 80 % erreicht wurden und Albers et al. (2013) legte dar, dass nur 6 % der Hüften nach PAO alle radiologischen Zielparameter (totale, anteriore, posteriore, laterale femorale Überdachung, Extrusionsindex, Tönnis-Winkel) erfüllen konnten [12, 13]. Die Rezidivrate der Hüftluxation bei diagnostizierter CP und dysplastischen Hüften mit progredienter Subluxation liegt in der aktuellen Literatur auch nach initial erfolgreichen postoperativen radiologischen Ergebnissen bei bis zu 23 % [14].

Mit zunehmender Verfügbarkeit werden CT-basierte 3‑D-Rekonstruktionen und Simulationen wertvolle Werkzeuge zur Verbesserung dieser Probleme. Sie ermöglichen bei komplexen multiplanaren Rekonstruktionen eine individualisierte Planung von Osteotomieverläufen, Korrekturwinkeln und Fragmentrotationen zur Optimierung der endgültigen Lastverteilung [15]. Eine konsekutive intraoperative Umsetzung kann mittels patientenspezifischer, 3‑D-gedruckter Schnittblöcke, Rotations- und Repositions-Guides oder intraoperativer 3‑D-Navigation von Fragmenten oder chirurgischen Instrumenten erfolgen. Erste Studien konnten zeigen, dass beide Ansätze – einzeln oder kombiniert angewendet – die Operations- und Durchleuchtungszeiten, den Blutverlust und die Rate epiphysärer Schäden reduzieren bei mindestens gleichwertigen klinisch-radiologischen Ergebnissen [16–20]. Hinsichtlich Präzision zeigte Sallent et al. (2017) im experimentellen Beckenmodell durch den Einsatz von Guides gegenüber der Freihandtechnik eine Verbesserung um 6–10 mm, mit dem Anteil an Osteotomien mit Abweichungen > 10 mm bei 0 % verglichen mit 27 % Freihand [21].

Während eine Effizienz- und Präzisionssteigerung für jeden operativen Eingriff erstrebenswert ist, sehen wir deren klinische Mehrwert aktuell insbesondere bei komplexen, multiplanaren Rekonstruktionen und vulnerablen Patientengruppen. Für die pädiatrische Population fehlen bislang systematische klinische Studien die den Nutzen von 3‑D-Applikationen untersuchen und somit eine evidenzbasierte Indikationsstellung ermöglichen. In der vorliegenden Arbeit präsentieren wir drei Fallbeispiele, um das Potenzial der 3‑D-Rekonstruktion in der präoperativen Planung, der intraoperativen Umsetzung mittels patientenspezifischen Führungs-Guides aufzuzeigen bei komplexer posttraumatischer Rekonstruktion sowie im Kontext der angeborenen und sekundären neurologisch bedingten dysplastischen Hüfte im Kindes- und Jugendalter aufzuzeigen.

## Methoden

Diese Arbeit ist eine retrospektive Fallanalyse von drei Patienten. Zwischen 2023 und 2025 erhielten sie an zwei Referenzzentren eine Hüftgelenksrekonstruktion mittels Becken- und/oder Femurosteotomie. Ziel der Fallselektion war es, eine möglichst vielfältige Anwendung der verfügbaren Modalitäten bei unterschiedlichen Indikationen im Rahmen eines etablierten klinischen Workflows aufzuzeigen, ohne Anspruch auf einen vergleichenden Wirksamkeitsnachweis. Die perioperative Planung sollte daher folgende Kriterien erfüllen: Die Nutzung von CT-basierten 3‑D-Rekonstruktionen in der präoperativen Planung und/oder der intraoperativen Umsetzung („computer-assisted research and development“ [CARD]) sowie ein postoperatives Follow-up von mindestens 9 Monaten mit konventionell-radiologischen Verlaufskontrollen. Die präoperative CARD-Analyse und die Herstellung der patientenspezifischen Guides erfolgten extern. Die intraoperative Technik entsprach etablierten Beschreibungen und klinikinternen Standards. Alle Eingriffe wurden von erfahrenen Kinderorthopäden mit über 10 Jahren Erfahrung durchgeführt oder supervidiert. Patientendaten wurden aus dem elektronischen Patientenarchiv und dem Bildarchivierungssystem extrahiert und anonymisiert. Aufgrund des retrospektiven Charakters und der Anonymisierung der Daten entfiel die individuelle Einwilligungspflicht.

## Fall 1: Beidseitige Hüftrekonstruktion mit Beckenosteotomie nach Dega und VDRO, rechts CARD-geplant

**Abb. 1:**
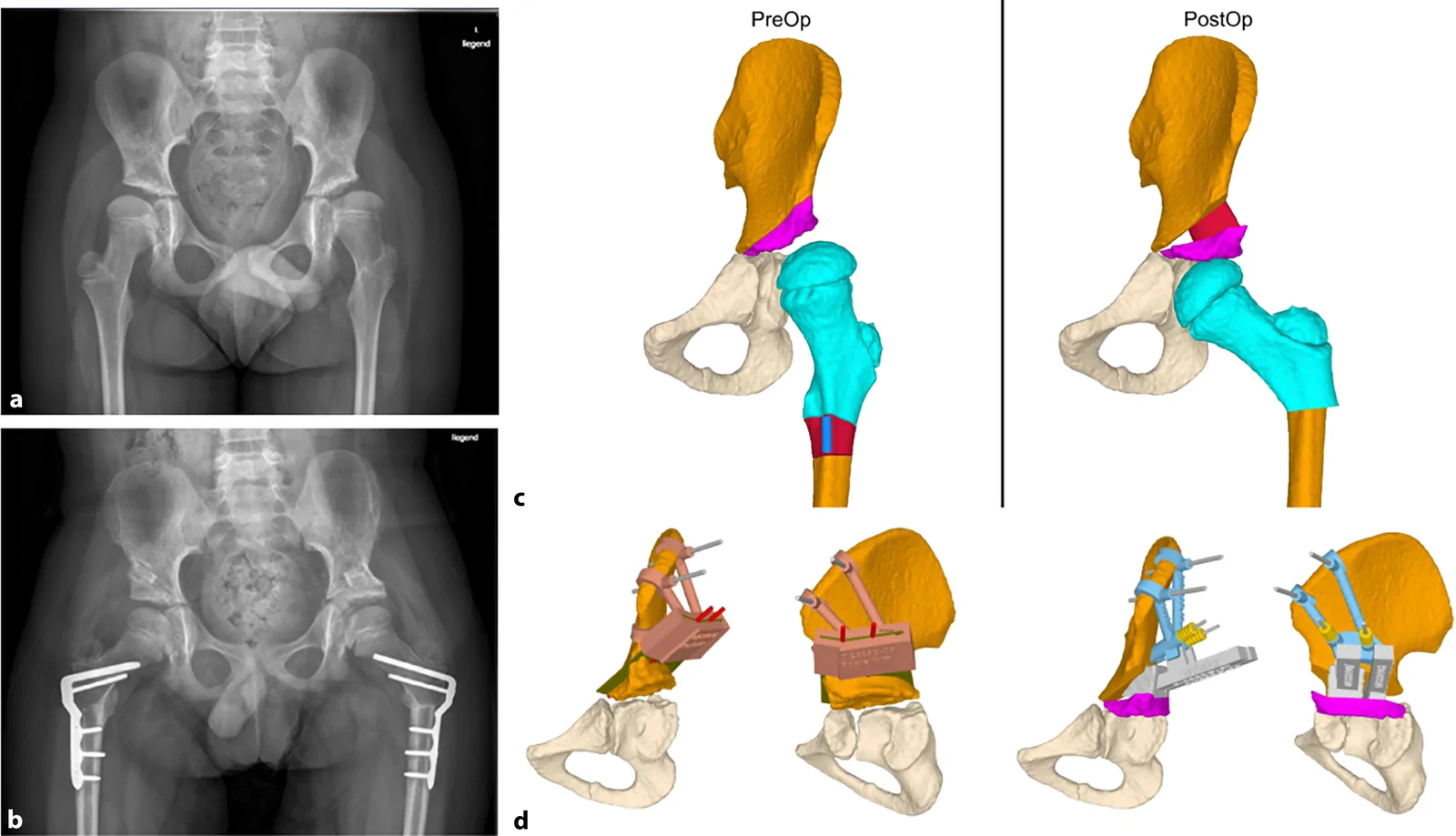
**a** Präoperative a. p. Röntgenaufnahme **b** 12 Monate postoperative a. p. Röntgenaufnahme **c** Visualisierung der prä- und postoperativen anatomischen Architektur **d** Visualisierung der intraoperativen Guides

Ein 11-jähriger Junge mit beidseitiger neurogener Hüftdysplasie und progredienter Hüftsubluxation bei Zerebralparese (GMFCS IV) und damit verbundener muskulärer Hypotonie und Hyperlaxizität. Konventionell-radiologisch zeigte sich eine Hüftdysplasie mit Azetabulumwinkel links 28°, rechts 17° sowie einem Migrationsindex (MI) von links 47 %, rechts 31 % (Abb. 1a). In der klinischen Untersuchung präsentierte er ein erweitertes Bewegungsausmaß („range of motion“ [ROM]) der Hüfte bei Innen‑/Außenrotation in Hüftstreckung rechts 80-0-100°, links 90-0-40°, in Hüftflexion rechts 90-0-70°, links 100-0-40° und Abduktion in Hüftstreckung bds. 90°, in Hüftflexion bds. 40°.

Aufgrund der Progredienz lag die Indikation für eine beidseitige Hüftrekonstruktion mit Beckenosteotomie nach Dega und VDRO vor. Anhand der in der klinischen Routine durchgeführten CT-Untersuchung erfolgte eine präoperative CARD-Analyse und -Planung (Abb. 1c) mit der Herstellung von intraoperativen Guides für die linke Seite, während rechts konventionell geplant und durchgeführt wurde. Centrum-Collum-Diaphysen-Winkel (CCD) rechts unter BV: 145°, links nach CARD: 154,3°. Femorale Antetorsion (FAT) rechts im CT 44°, links nach CARD 63,4°. Das Korrekturziel bds. lag bei einem CCD von 125° und einem FAT von 15°.

Zunächst erfolgte rechts das konventionelle Vorgehen nach etablierten Methoden: VDRO mit Klingenplatte (Locking Cannulated Blade (LCB), Child 3,5 mm, Klingenlänge 50 mm, Offset 6 mm, OrthoPediatrics Corp., Warsaw, IN, USA) über Subvastus-Zugang am proximalen Femur. Korrekturkontrolle mittels Rotationsdrähten und BV. Beckenosteotomie nach Dega mit Korrekturkontrolle mittels BV über eine Hautinzision von circa 10 cm, lateral der Beckenkammapophyse, ausgehend von der Spina iliaca anterior superior und nach dorsal ziehend. Spaltung der Apophyse und stumpfe subperiostale Ablösung der Abduktorenmuskulatur vom Ilium. Markierung der Osteotomiehöhe unter BV mittels Kirschner-Draht. Nach vorhergehender Osteotomie der Kortikalis entlang der geplanten Osteotomie nach Dega mit geradem 10 mm Lambotte-Meißel Komplettierung der Osteotomie mit gebogenem 10 und 15 mm nachfolgendem Meißel. Einbringen eines Knochenblockes nach vorheriger Einstellung der gewünschten Korrektur mittels Spreizer.

Links die Umsetzung der CARD-Planung wie folgt (Abb. 1d): Einbringen eines ersten Guides zum Setzen der Rotations- und Plattenführungsdrähte. Über die liegenden Drähte Wechsel zum Schnittblock zur biplanaren Osteotomie des Femurs mit Keilexzision. Es folgt die Korrektur durch Einbringen einer analogen Klingenplatte über die vorgesetzten Drähte, danach Überprüfung der Korrektur klinisch und mittels BV. Austausch der Drähte gegen die jeweilige Schraube. Am Becken gleiches Zugehen wie konventionell. Anbringen des Schnittblockes nach vorheriger Darstellung der Spina iliaca anterior superior und des Übergangs zur Gelenkskapsel. Der Block wird durch zwei Drähte über den Beckenkamm fixiert. Nach erfolgter Osteotomie, Wechsel auf den Korrektur-Guide über die einliegenden Drähte. Der Guide gewährleistet die Korrektur durch zwei separate darin integrierte Spreizer. Anpassen des zuvor femoral entnommenen Knochenblockes mittels separaten Guides. Bei liegendem Korrektur-Guide wird über die gleichen Drähte ein weiterer Guide eingebrachten zur Entnahme eines zusätzlichen definierten Knochenblockes. Schrittweises Ersetzen der beiden Spreizer durch den vorbereiteten femoralen Knochenblock und des zusätzlichen Knochenblock dorsal davon. Überprüfung beider Korrekturen klinisch und mittels BV. Operationsdauer 209 min, Strahlungszeit 0:47 min, Blutverlust 400 ml.

Das postoperative Prozedere entsprach den Standards des konventionellen Vorgehens bis auf ergänzende CT-Untersuchungen. Zur Überprüfung des postoperativen Ergebnisses im Vergleich zur präoperativen Planung nach CARD erfolgte eine Woche postoperativ eine erste radiologische Verlaufskontrolle mittels CT von Becken und Oberschenkel sowie Röntgen-Beckenübersicht. Diese zeigten regelrechte Stellungsverhältnisse der intraoperativ wiederhergestellten Hüftzentrierung (CCD rechts 127°, links 125°, FAT rechts 18°, links 11°) und vollständige Überdachung. Eine regulär 6 Wochen postoperativ erneut durchgeführte CT-Untersuchung diente zum Ausschluss eines sekundären Korrekturverlustes der azetabulären Korrektur durch eine allfällige Nachsinterung der Knochenblöcke, wie auch zur Dokumentation der Konsolidation für die Belastungsfreigabe. Weitere Verlaufskontrollen erfolgten nach 6 und 12 Monaten konventionell-radiologisch (Abb. 1b). Diese zeigten weiterhin regelrechte Korrekturverhältnisse (CCD bds. 128°, MI rechts 12 %, links 15 %) bei klinisch symmetrischer, normalisierter ROM: Außen‑/Innenrotation in Hüftstreckung 35-0-40° beidseits, in Hüftbeugung 60-0-50° beidseits. Abduktion in Hüftstreckung 50° beidseits, in Hüftbeugung 65° beidseits.

## Fall 2: Reverse PAO

**Abb. 2:**
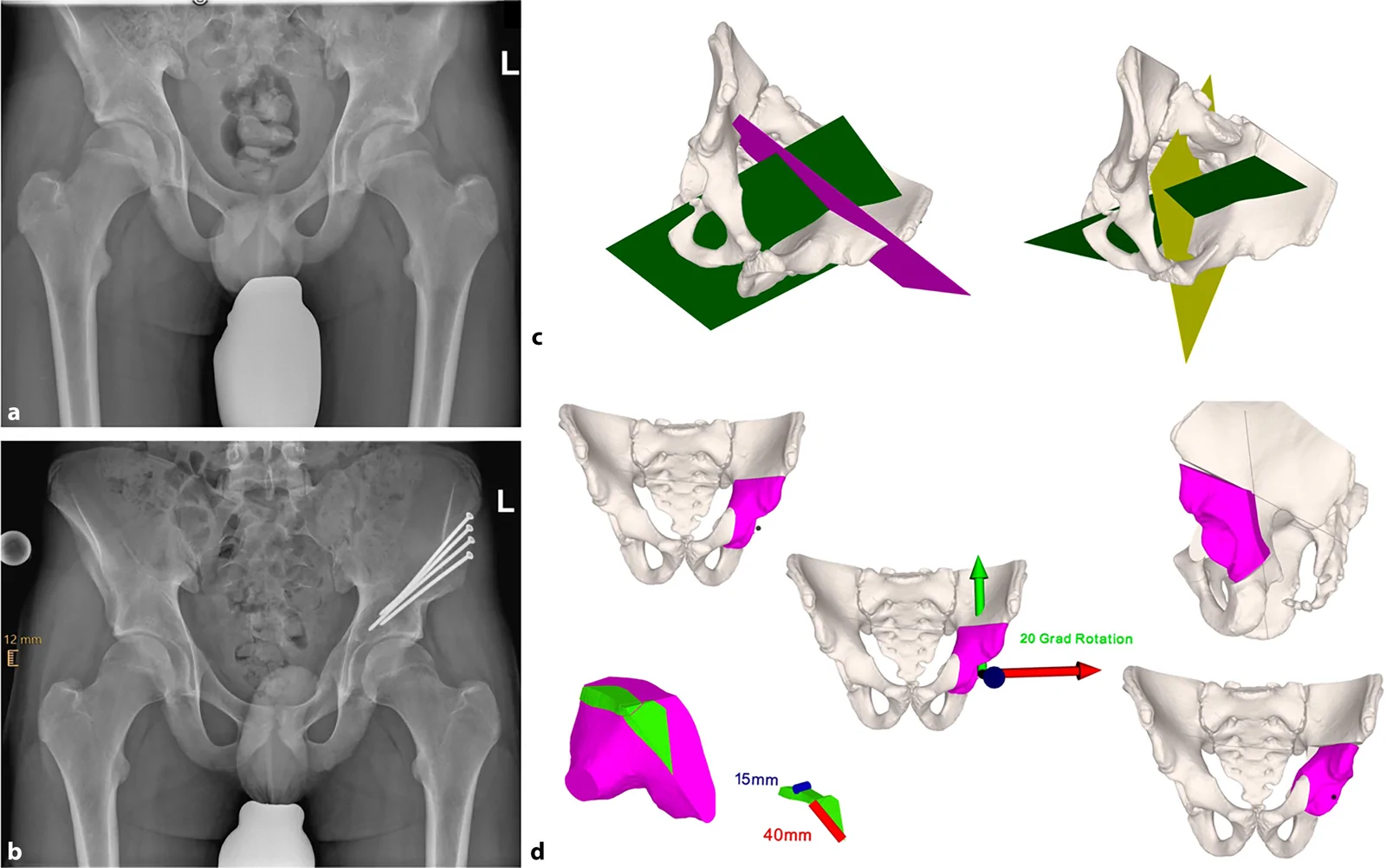
**a** Präoperative und **b** 12 Monate postoperative a. p. Röntgenaufnahme **c** Visualisierung der Osteotomieebenen **d** Visualisierung der Reorientierung sowie des Fragmentes inklusive Verschnitt

Ein 15-jähriger Junge mit zunehmenden Leistenschmerzen für 18 Monate, vor allem beim Sport und beim längeren Sitzen, konservativ ausbehandelt. Bei V. a. eine Pfannenretrotorsion durch ein „crossing-sign“ im konventionellen Röntgenbild (Abb. 2a) erfolgte eine weitergehende Diagnostik mittels Torsions-MRT. Bei bestätigter Retrotorsion von 3° links und 0° rechts erfolgte eine präoperative CARD-Analyse zur Simulation des intraoperativen knöchernen Konflikts bei Korrektur der azetabulären Retrotorsion mit einer Zielanteversion des Azetabulums von 15°.

Intraoperativ wurde die Analyse zur Referenzierung der Osteotomieebenen (Abb. 2c), zur Entfernung des knöchernen Überstandes des Fragmentes und freien Rotationsmöglichkeit des Aztetabulums (Abb. 2d) genutzt bei sonst etabliertem Vorgehen. Die Operationszeit betrug 135 min, Röntgenzeit 2:23 min, Blutverlust 430 ml.

Die klinisch-radiologische Kontrolle 18 Monate postoperativ (Abb. 2b) zeigte eine gute Pfannenüberdachung, ein präoperativ sichtbares „crossing-sign“ war im konventionellen Beckenübersichtsröntgen postoperativ nicht mehr ersichtlich. Eine CT-Untersuchung zur Verifizierung der geplanten Korrektur ist bei mangelnder therapeutischer Konsequenz bei regredienten Beschwerden und im Alltag beschwerdefreiem Patienten nicht erfolgt.

## Fall 3: CARD-geplante femorale Korrekturosteotomie und Implantation Verlängerungsmagnetnagel proximales Femur rechts

**Abb. 3:**
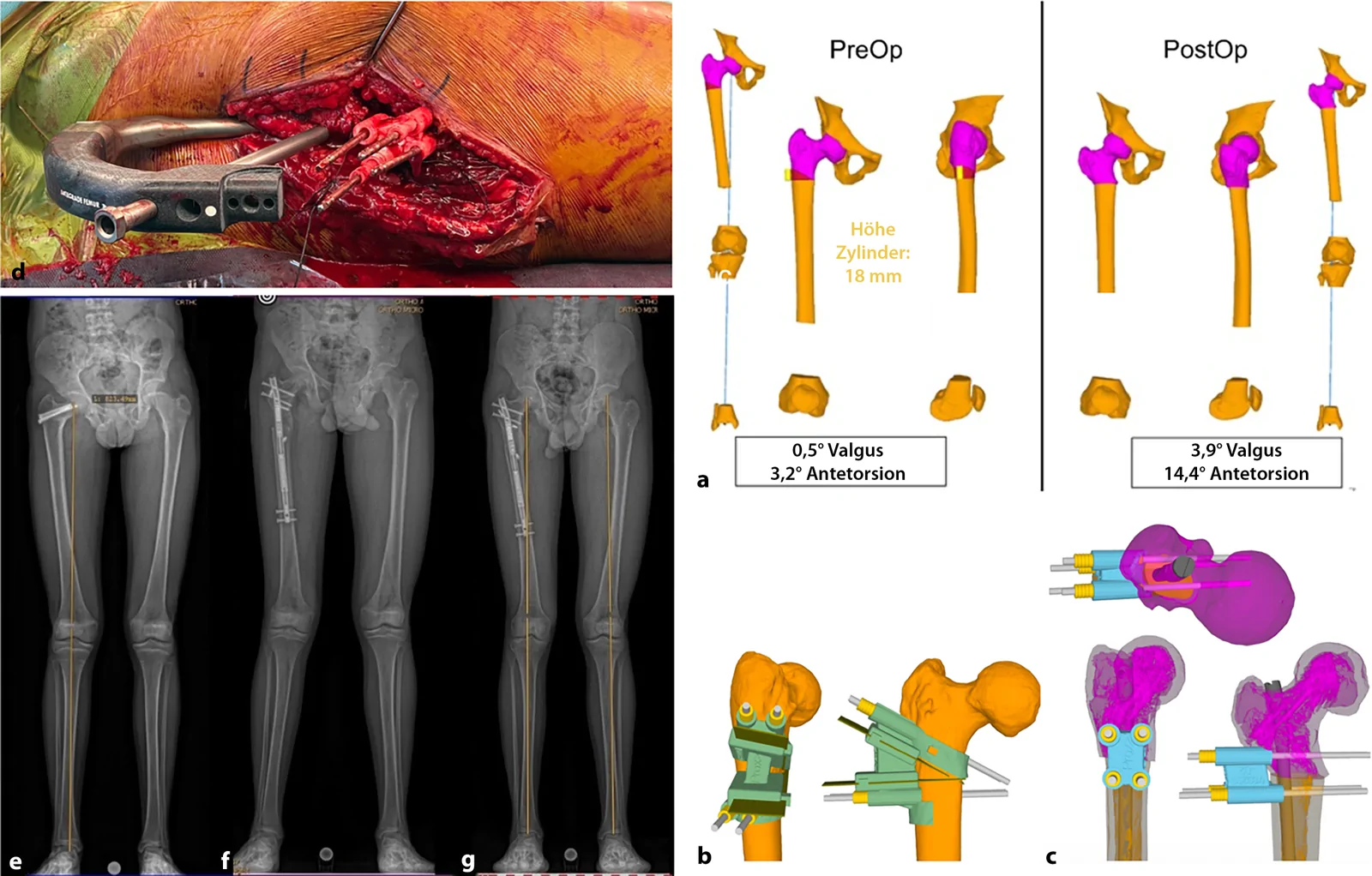
**a** Visualisierung der prä- und postoperativen Achsen und Rotationen **b** Visualisierung des Schnittblocks **c** Visualisierung des Korrektur-Guides **d** Intraoperative Aufnahme mit Korrektur-Guide und eingebrachtem Precise-Nagel **e** präoperatives a. p. Orthoradiogramm und Verlaufskontrollen nach **f** 8 Wochen und **g** 8 Monaten

Ein 14-jähriger Patient mit mehrdimensionaler Fehlstellung der rechten Hüfte posttraumatisch nach initialer Versorgung mittels kanülierter Schraubenosteosynthese einer Schenkelhalsfraktur in einer anderen Klinik. Zusätzlich bestand eine symptomatische Beinlängendifferenz von 43 mm zu Ungunsten rechts (Abb. 3c). In der klinischen Untersuchung präsentierte sich der Patientin mit einem hinkenden Gangbild bei einem ROM der Hüfte von 90-0-0° in Flexion/Extension sowie 30-0-20° in Innen‑/Außenrotation.

Aufgrund der Symptomatik stellten wir die Indikation zur Korrekturosteotomie mit Verlängerung über einen Precise-Nagel. Es erfolgte eine präoperative CARD-Analyse, diese zeigte rechts ein FAT von 3,2° und einen CCD von 101,1°. Unter Einbezug der Gegenseite konnte die exakte Korrektur modelliert und geplant werden (Abb. 3a).

Intraoperativ erfolgte die biplanare Femurosteotomie mit Keilexzision mittels unter Röntgenkontrolle gesetztem Schnittblock. Anschließend der Wechsel auf den Repositions-Guide vor Einbringen des Magnetnagels (Precice Nail 210° Trochanteric Blend, 12,5 × 245 mm, NuVasive Specialized Orthopedics, Aliso Viejo, CA, USA; distale Bolzen 2 × 32,5 mm, 3 × anteriore Pollerschrauben) zum Halten der geplanten Korrektur bis zum Verriegeln desselben (Abb. 3b). In der intraoperativen Abschlussdokumentation konnte die gewünschte Korrektur bestätigt werden. Operationsdauer 148 min inklusive Entfernung des bereits einliegenden Osteosynthesematerials, Blutverlust 500 ml, Strahlenexposition 2:56 min.

Eine Woche postoperativ erfolgte die radiologische Kontrolle von Becken und Femur. Weitere radiologischen Kontrollen zur Dokumentation der Segmenttrennung am Femur und Beinlänge erfolgten 2, 4 und 8 Wochen (Abb. 3d) postoperativ, welche eine regelrechte Stellung mit einem CCD von 123° und zeitgerechte Kallusbildung zeigten. In den weiteren Kontrollen 5 und 8 Monate postoperativ zeigte sich ein regelrechter klinisch-radiologischer Verlauf mit flüssigem Gangbild bei ausgeglichener Beinachse und -länge (rechts 1° Valgus bei 864 mm, links 4° Valgus bei 870 mm, Abb. 3e) und bds. zentrierten Hüftköpfen.

## Diskussion

Diese Fallserie illustriert die Möglichkeiten präoperativer 3‑D-Planung und ihre klinische Umsetzung mittels CARD-Technologie bei komplexen kindlichen und jugendlichen Hüftfehlstellungen unterschiedlicher Genese (angeborene und sekundäre neurologische Hüftdysplasie, posttraumatische Fehlstellung). In allen Fällen konnte eine reproduzierbare intraoperative Umsetzung der geplanten Korrekturwinkel erreicht und eine stabile Verbesserung radiologischer Parameter (CCD-Winkel, MI, FAT) erzielt werden. Weitere intraoperative Parameter wie Operationsdauer, Strahlenexposition und Blutverlust lagen in den klinikinternen Normbereichen.

Fall 1, beidseitige Hüftrekonstruktion mit Beckenosteotomie nach Dega und VDRO, erlaubt durch die Kombination aus konventioneller (rechts) und CARD-gestützter Planung (links) bei einem Patienten mit sekundärer neurologischer Hüftdysplasie und progredienter Hüftsubluxation einen intraindividuellen Vergleich. Die beidseits erreichten radiologischen Zielparameter bestätigen die Möglichkeit der präzisen Umsetzung der CARD-Planung bei einer häufigen Indikation und Routineeingriff. Fall 2, reverse PAO, unterstützt nebst der präoperativen Planung die intraoperative Referenzierung zur Entfernung des knöchernen Überstandes des Fragmentes und somit die freie Rotationsmöglichkeit des Azetabulums. Fall 3, eine kombinierte Korrekturosteotomie und Beinverlängerung mittels magnetischem Marknagel bei einer posttraumatischen Deformität, zeigt, dass die präzise Planung und Durchführung einer 3‑D-Hüftkorrektur, quantifiziert mittels CCD und FAT-Winkel, mit weiteren operativen Schritten und Implantaten kombiniert werden kann. Aufgrund der effizienteren Planungs- und Behandlungsmöglichkeiten wird der Leidensdruck der Patienten reduziert und die Risiken zusätzlicher Operationen vermieden. Die Anwendungen augmentieren unterschiedliche, etablierte Vorgehen.

### Aktuelle Studienlage

Unterschiedliche Arbeitsgruppen erforschen den Einsatz von 3‑D-Bildgebung und -Navigation im Kontext von Hüftrekonstruktionen bei DDH mit Fokus auf die klinische Anwendung bis hin zu experimentellen Augmented-Reality‑, Robotik-Navigations- und minimalinvasiven Ansätzen in Kadaverstudien [22–24]. Dabei liegt der Schwerpunkt der aktuellen Studienlage bei PAO der adulten Population. Imai et al. (2020) konnte zeigen, dass der Einsatz der 3‑D-Navigation zu verbesserten Endresultaten führte im Gegensatz zur konventionellen Gruppe [16]. Lee et al. (2025) integrierte Finite-Elemente-Analysen in die Planung und erreichte mit 3‑D-gedruckten intraoperativen Guides eine sehr hohe Präzision der Umsetzung der geplanten Korrektur [17]. In der pädiatrischen Patientenpopulation wurde durch die Verwendung von Führungsblöcken bei mit konventionellen Methoden vergleichbaren radiologischen Endergebnissen und unveränderten McKay- und/oder Severin-Scores postoperativ insbesondere die Verbesserung der intraoperativen Belastung durch kürzere Operations- und Durchleuchtungszeiten und geringerem Blutverlust festgestellt: Zheng et al. (2017) und Sun et al. (2023) für proximale Femurosteotomien sowie Yu et al. (2021) für Salter-Beckenosteotomien [18–20]. Eine 2024 publizierte Studie von Incze-Bartha et al. demonstriert die Relevanz von CT-basierten 3‑D-Rekonstruktionen und Simulationen mit Finite-Elemente-Analysen in der präoperativen Planung für Kinder [15]. Die Heterogenität dieses geringen Evidenzbestands lässt keine direkte Vergleichbarkeit mit unserer Fallserie zu. Unsere Ergebnisse schließen sich jedoch den berichteten Erfolgen an, bei operativen Ergebnissen im Zielbereich und intraoperativen Parametern, die zentrumsinternen Standards entsprechen.

### Umsetzbarkeit, Kosten und Systemkontext

Die praktische Umsetzbarkeit der 3‑D-Planung ist eng an organisatorische und strukturelle Voraussetzungen gebunden. In unserem Setting ermöglicht die enge Zusammenarbeit mit einem universitären Forschungszentrum sowie spezialisierten Industriepartnern eine weitgehende Standardisierung des Planungs- und Produktionsprozesses. Hierdurch können präoperative Bildgebung, virtuelle Planung, Validierung und Herstellung patientenspezifischer Guides in einem definierten Zeitfenster erfolgen. Die durchschnittliche Dauer vom initialen Planungsschritt bis zur operativen Umsetzung beträgt in unserem Ablauf etwa 6 Wochen. Daraus ergibt sich, dass diese Verfahren primär für elektive Korrekturen geeignet sind. Akut-traumatologische oder onkologische Indikationen mit zeitkritischem Operationsbedarf sind für dieses Vorgehen nicht geeignet. Die Indikationsstellung ist hinsichtlich der Kostenstruktur ein relevanter Aspekt bei der Implementierung 3‑D-gestützter Verfahren. Für Kinder mit angeborenen oder neuroorthopädischen Erkrankungen besteht in diesem Land ein Zugang zu staatlichen Unterstützungsleistungen, dies trifft nicht universell auf alle Gesundheitssysteme zu. Die direkten Zusatzkosten der 3‑D-Planung (Bildgebung, virtuelle Planung und patientenspezifische Guides, Kostenpunkt im oben geschilderten Fall aufgrund von Studienzwecken 2400 Fr., im klinischen Alltag 4800 Fr.) stehen in Zukunft potenziellen Einsparungen durch reduzierte Operationszeiten und insbesondere der möglichen Reduktion von Revisionsoperationen durch eine erhöhte Präzision bei geringerer Strahlungsdosis gegenüber. Diese Annahme ist in einem etablierten Setting zu prüfen, bei welcher innert der Lernkurve bereits ein gewisses Plateau erreicht wurde.

### Limitationen

Das retrospektive Design dieser Arbeit als Fallbericht mit lediglich drei Fällen birgt relevante Limitationen, insbesondere durch Selektions-Bias, fehlende Vergleichsfälle sowie nicht standardisierte Nachuntersuchungen. Daher sind keine generalisierbaren Aussagen zur klinischen Relevanz möglich. Die bewusst gewählte breite Variabilität der Indikationen und operativen Verfahren erlaubt zudem keine vergleichenden Analysen. Hinsichtlich der Korrekturquantifizierung ist zudem dem Umstand Rechnung zu tragen, dass das Korrekturziel im präoperativen 3‑D-CT definiert wurde, postoperativ aufgrund der Strahlenbelastung jedoch lediglich eine konventionell-radiologische Bildgebung erfolgte.

### Perspektiven und Forschungsbedarf

Die präsentierten Fälle belegen die erfolgreiche Umsetzung unterschiedlicher 3‑D-geplanter Anwendungen. Aktuelle Studien zur Implementierung dieser Techniken bei der PAO zeigen eine positive Tendenz hinsichtlich Präzision und Machbarkeit. Zur Bestätigung der klinischen Relevanz sowie der Langzeitergebnisse sind jedoch weitere Studien erforderlich. Diese sollten idealerweise in einem prospektiven, multizentrischen Design mit größeren Fallzahlen geplant werden. Ein systematischer Vergleich zwischen konventioneller 2‑D-Planung und -Umsetzung sowie neuen, 3‑D-basierten Verfahren sollte nebst intraoperativen Parametern und dem langfristigen Korrekturerfolg inklusive Rezidivrisiko insbesondere auch Aufschluss über Veränderungen der gesundheitsbezogenen Lebensqualität geben. Im Hinblick auf planerischen Mehraufwand und zusätzliche Kosten ist eine weitere, differenzierte Analyse verschiedener Patientengruppen sinnvoll. Dadurch könnten Subpopulationen identifiziert werden, die einen klinisch relevanten Zusatznutzen aufweisen und möglicherweise besonders von einer Behandlung in Zentren profitieren, die 3‑D-Applikationen nutzen. Dies könnte zur Weiterentwicklung evidenzbasierter Versorgungsalgorithmen beitragen. Im Kontext dieser Forschungsfragen ist auch die Rolle der chirurgischen Expertise zu berücksichtigen. Eine Korrelation der Outcomes von konventionellen und 3‑D-gestützten Vorgehen mit der operativen Erfahrung der Chirurg:innen könnte determinieren, ob insbesondere weniger erfahrene Operateur:innen durch diese Techniken bessere Ergebnisse erzielen und ob sich Effekte bezüglich Ausbildung und Lernkurve zeigen. 3‑D-Modelle und patientenspezifische Guides können als wirkungsvolles Trainingsinstrument fungieren. Dennoch sind diese nur ergänzend und nicht als Ersatz für die klassische Planung und Freihandumsetzung zu sehen, welche ein fundiertes Verständnis der anatomischen Verhältnisse und biomechanischen Belastungen voraussetzt. Insbesondere ist zu beachten, dass die ungenaue oder inkorrekte Anwendung dieser Hilfsmittel zu gravierendem Therapieversagen führen kann.

## Fazit für die Praxis

- Diese Fallserie zeigt, wie Routineeingriffe als auch die Versorgung komplexer Einzelfälle durch prä- und intraoperative Anwendung 3‑D-basierter Rekonstruktionen im Sinne patientenspezifischer, individualisierter Medizin weiterentwickelt werden können.
- Ob diese Techniken zum langfristigen Ziel der Steigerung gesundheitsbezogener Lebensqualität durch verbesserte Korrekturergebnisse beitragen können, bleibt Gegenstand des aktuellen Forschungsbedarfs.

## Data Availability

Alle in dieser Fallserie analysierten Daten sind in diesem veröffentlichten Artikel enthalten.

## Abkürzungen

BV: Bildverstärker
CARD: „Computer-assisted research and development“
CCD: Centrum-Collum-Diaphysen-Winkel
CP: Zerebralparese
DDH: „Developmental dysplasia of the hip“
FAI: Femoroazetabuläres Impingement
FAT: Femorale Antetorsion
GMFCS: Gross Motor Functions Classification System
MI: Migrationsindex
PAO: Periazetabuläre Osteotomie
ROM: „Range of motion“
VDRO: Varisierende, derotierende proximale Femurosteotomie
